# Myofibroblast androgen receptor expression determines cell survival in co-cultures of myofibroblasts and prostate cancer cells *in vitro*

**DOI:** 10.18632/oncotarget.24913

**Published:** 2018-04-10

**Authors:** Helen M. Palethorpe, Damien A. Leach, Eleanor F. Need, Paul A. Drew, Eric Smith

**Affiliations:** ^1^ Discipline of Surgical Specialities, The University of Adelaide, Basil Hetzel Institute for Translational Health Research, The Queen Elizabeth Hospital, Woodville South, Australia; ^2^ Department of Surgery and Cancer, Imperial College London, London, United Kingdom; ^3^ School of Nursing and Midwifery, Flinders University, Adelaide, Australia; ^4^ Molecular Oncology, Basil Hetzel Institute for Translational Health Research, The Queen Elizabeth Hospital, Woodville South, Australia

**Keywords:** androgen receptor, direct co-culture, indirect co-culture, myofibroblast, prostate cancer

## Abstract

Fibroblasts express androgen receptor (AR) in the normal prostate and during prostate cancer development. We have reported that loss of AR expression in prostate cancer-associated fibroblasts is a poor prognostic indicator. Here we report outcomes of direct and indirect co-cultures of immortalised AR-positive (PShTert-AR) or AR-negative (PShTert) myofibroblasts with prostate cancer cells.

In the initial co-cultures the AR-negative PC3 cell line was used so AR expression and signalling were restricted to the myofibroblasts. In both direct and indirect co-culture with PShTert-AR myofibroblasts, paracrine signalling to the PC3 cells slowed proliferation and induced apoptosis. In contrast, PC3 cells proliferated with PShTert myofibroblasts irrespective of the co-culture method. In direct co-culture PC3 cells induced apoptosis in and destroyed PShTerts by direct signalling. Similar results were seen in direct co-cultures with AR-negative DU145 and AR-positive LNCaP and C4-2B prostate cancer cell lines. The AR ligand 5α-dihydrotestosterone (DHT) inhibited the proliferation of the PShTert-AR myofibroblasts, thereby reducing the extent of their inhibitory effect on cancer cell growth.

These results suggest loss of stromal AR would favour prostate cancer cell growth *in vivo*, providing an explanation for the clinical observation that reduced stromal AR is associated with a poorer outcome.

## INTRODUCTION

Androgens are essential for the normal development of the prostate, and, in the adult, are required for prostate epithelial cell survival and function. In the early phases of prostate development the androgen receptor (AR) is expressed exclusively in mesenchymal cells, which in turn regulate epithelial cell growth and differentiation, and thereby prostate size [1]. In the adult prostate, AR is expressed in both stromal and epithelial compartments [2, 3]. Here androgens help maintain stromal smooth muscle and epithelial differentiation and function via reciprocal stromal-epithelial cell interactions [2].

Androgens and AR also play a pivotal role in the development and progression of prostate cancer. The majority of studies investigating the role of AR in prostate cancer have focused on its function in the malignant epithelial cells, however it is becoming increasingly apparent that androgen signalling in the stroma can also influence cancer development and progression.

The stroma of the normal prostate is comprised predominantly of smooth muscle cells, with a small number of fibroblasts and myofibroblasts. In prostate cancer, myofibroblasts, or cancer-associated fibroblasts (CAFs), are the predominant stromal cell type and influence the growth, invasiveness and metastasis of cancer cells [4–6]. The AR is strongly expressed in the stroma in early prostate cancer, but may be decreased in areas surrounding cancerous tissue, especially in androgen-independent cancer [7, 8], and this can be associated with early relapse [3]. We have shown a significant association between low AR levels in cancer-associated stroma and increased prostate cancer-related death at 1, 3, and 5 years post-diagnosis [5, 6]. High AR levels in the epithelial cells were associated with higher Gleason score and higher serum PSA levels, but not with outcome, whilst, in contrast, low AR levels in the stroma were associated with more extensive disease, and a greater risk of prostate cancer-related death [5]. Whilst this indicates that AR expression in the prostate stroma is an important prognostic biomarker [9–12], how AR influences cancer progression is unclear.

Fibroblasts have the potential to influence the behaviour of epithelial cells via soluble or non-soluble factors. Soluble factors, such as growth factors, are typically studied using indirect co-culture systems, such as transwell chambers, or conditioned culture medium (CCM). Insoluble factors, which include matrix or cell membrane molecules, are studied in direct co-cultures, usually where the epithelial cells are added onto established stromal cell monolayers. Studying the behaviour of cells in direct co-cultures is challenging because it is difficult to distinguish and analyse each cell type separately.

We have overcome this limitation by stably transducing red fluorescent protein (RFP) into stromal myofibroblasts, and green fluorescent protein (GFP) into epithelial cancer cells, allowing monitoring or measuring by fluorescence microscopy or flow cytometry. In this study, we have co-cultured the prostate cancer cell lines with a telomerase immortalized human prostate stromal myofibroblast cell line, that was either stably transduced with AR (PShTert-AR), or with empty expression vector and not expressing AR (PShTert), to determine the effect of myofibroblast AR expression on myofibroblast-prostate cancer cell interactions *in vitro*.

## RESULTS

### The fate of PC3 cells in co-culture depended on myofibroblast AR expression

**Figure 1:**
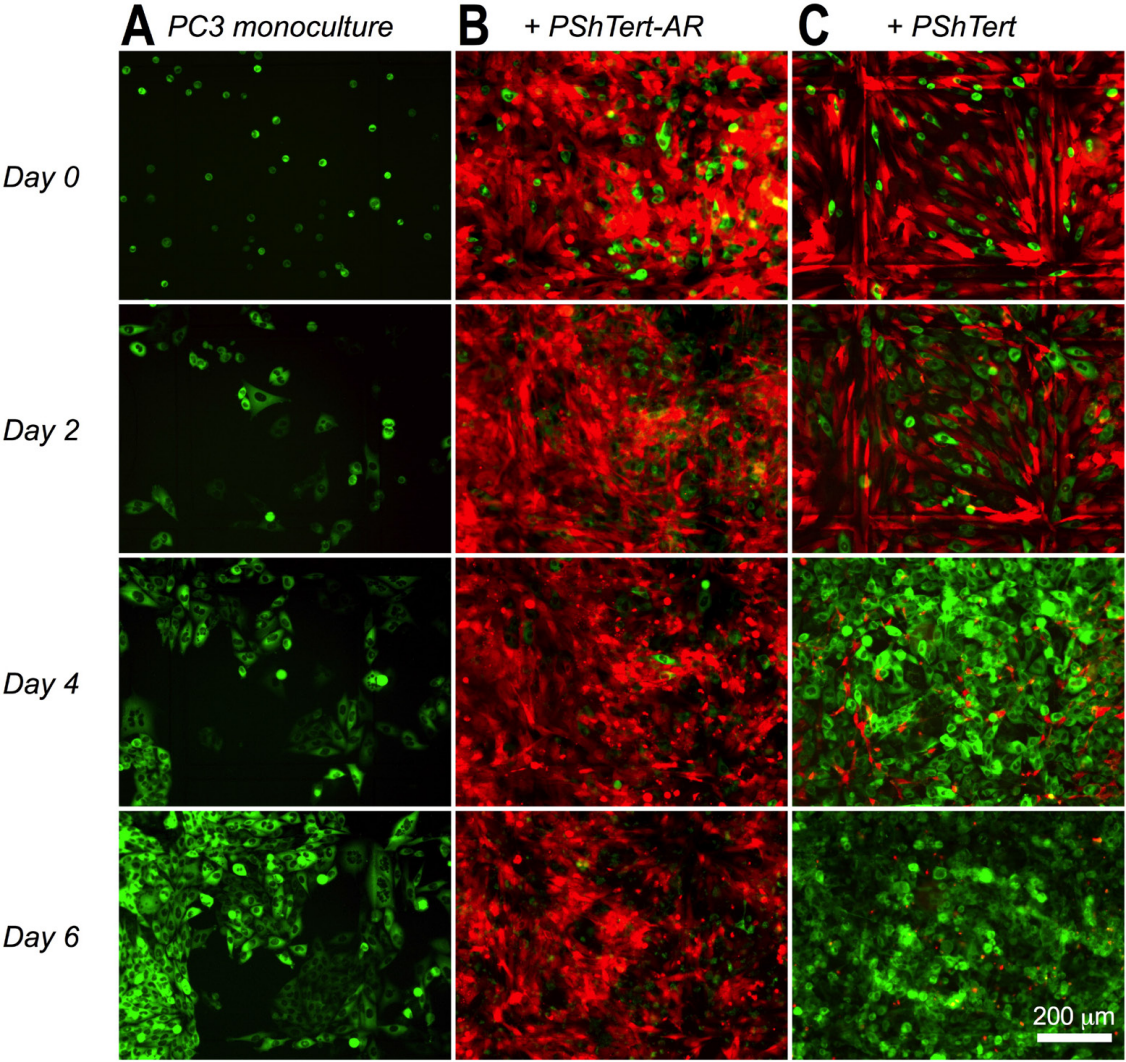
PC3 cells in monoculture and direct co-culture with myofibroblasts PC3 cells (GFP-labelled; 5 × 10^3^) were added to culture dishes with imprinted relocation grid (Ibidi) either in (**A**) monoculture or direct co-culture with 1.5 × 10^5^ RFP-labelled (**B**) PShTert-AR, or (**C**) PShTert myofibroblasts. Original magnification 100×.

The presence or absence of AR expression in the PShTert-AR and PShTert myofibroblasts and the PC3, LNCaP, C4-2B, and DU145 prostate cancer cells used in this study was confirmed by western immunoblot (Supplementary Figure 1). The growth of the AR-negative PC3 cells in direct co-culture with myofibroblasts was compared to that of cells in monoculture. After 6 days in monoculture the majority of PC3 cells were polygonal in shape, with distinct cell borders and minimal variation in size or shape. A fine perinuclear granulation was visible by phase contrast microscopy throughout the culture period. The PC3 cells were arranged singly or in small discohesive clusters on days 1 and 2, and then expanded in number to form cell aggregates, which ultimately coalesced into a cohesive sheet with well-defined cell borders by day 6 (Figure 1A).

**Figure 2:**
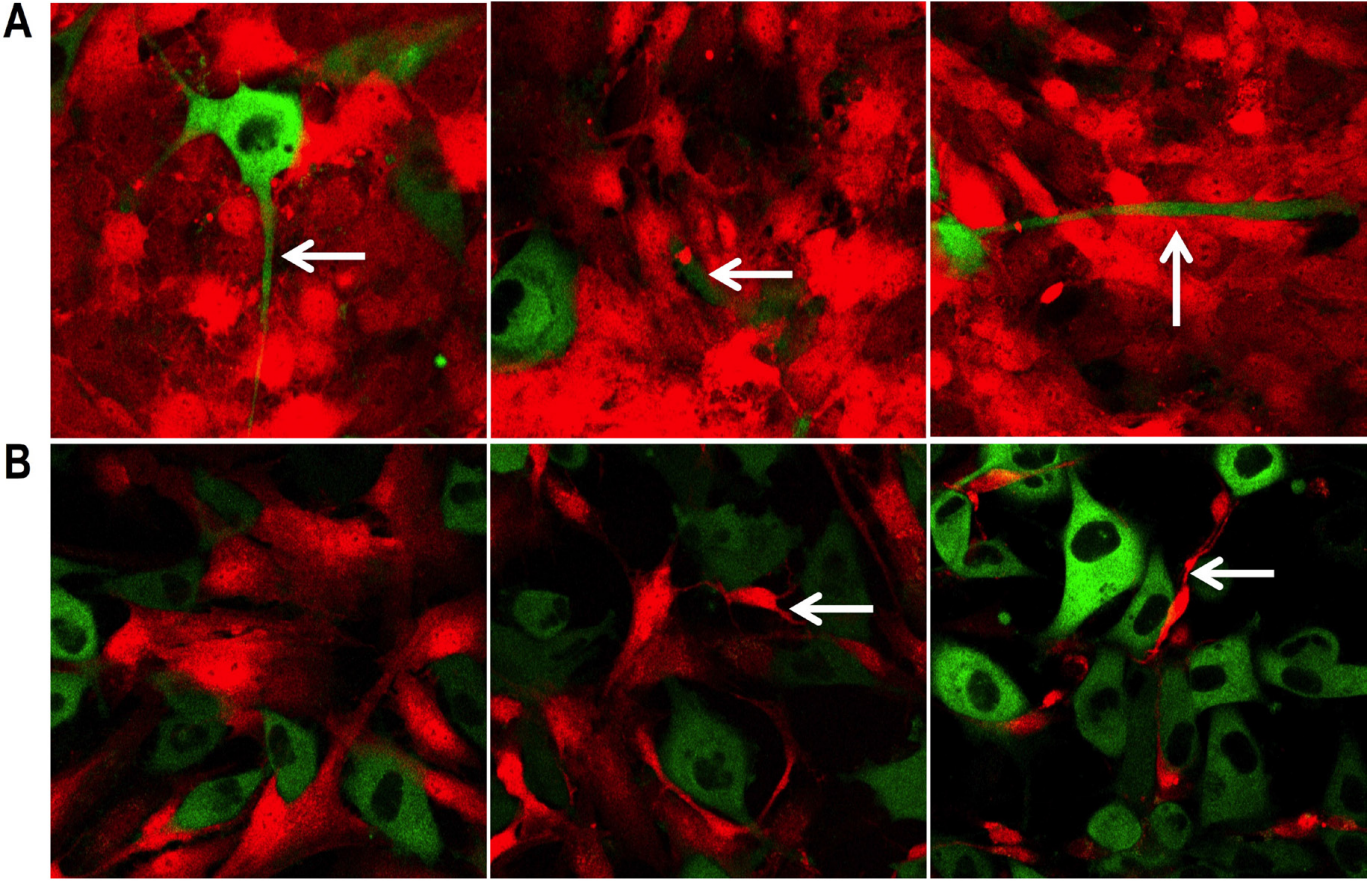
Specific morphological changes (**A**) Changes in PC3 cells directly co-cultured with PShTert-AR myofibroblasts. Arrows show extensions of the cytoplasm (left), cell disintegration (centre) and remnants of adherent extensions (right). (**B**) Progressive destruction of PShTert myofibroblasts directly co-cultured with PC3 cells.

The PC3 cells in direct co-culture with the PShTert-AR myofibroblasts were enlarged and pleomorphic within 24 hours, compared to the cells grown in monoculture. They formed short cytoplasmic extensions, which lengthened and narrowed by day 2 to 3, and failed to form the cohesive aggregates observed in monoculture. There was prominent cellular and nuclear shrinkage from day 2, followed by cell disintegration, leaving remnants of adherent extensions and cell fragments either attached to the well or free in the growth media (Figure 1B and Figure 2A).

The PC3 cells grown in direct co-culture with the PShTert myofibroblasts showed increased perinuclear granulation, together with cytoplasmic accumulation of numerous large, coarse granules from day 1. Short cytoplasmic extensions were observed from day 2 and these progressively narrowed and lengthened from days 3 to 6 as the cells proliferated. The number of PC3 cells increased rapidly, forming interconnected smallish rafts with clearing of the PShTert myofibroblasts immediately beneath. By day 6 the PC3 cells had formed large cohesive rafts of cells in the centre of the well (Figure 1C and Figure 2B).

### The fate of myofibroblasts in co-culture depended on their AR expression

The PShTert-AR myofibroblasts grown in direct co-culture with the PC3 cells retained the morphological features seen in monoculture. By 48 hours after seeding they were irregular in size and shape with a dense cytoplasm, and formed wide, cohesive bands of randomly orientated cells with occasional spaces between the bands (Figure 1B, day 0). This appearance did not change throughout the period of co-culture.

The PShTert myofibroblasts grown in direct co-culture with the PC3 cells retained the morphological features seen in monoculture in areas where there were no PC3 cells. There they grew as a relatively complete and uniform monolayer of narrow cells with clearly defined edges (Figure 1C, day 0). However, in areas underlying or immediately adjacent to PC3 cells, the PShTert myofibroblasts, over days, became condensed, elongated, irregularly shaped, and eventually disappeared. As the population of PC3 cells expanded, the numbers of PShTert myofibroblasts decreased significantly (Figure 1C and Figure 2B). The density and morphology of myofibroblasts remote from the PC3 cells appeared similar to that of cells in monoculture.

In confrontation assays the myofibroblasts and PC3 cells were separated by a 500 μm gap at the time of seeding (Supplementary Figure 2). The cells proliferated and migrated during culture, and the interactions were observed where the two cell fronts met. The fates of the cells in this assay were similar to those seen in direct co-cultures. For the PC3 cells and PShTert-AR myofibroblasts the gap closed relatively slowly, and where the migrating fronts met the morphology of the PC3 cells altered and their number reduced with time (Supplementary Figure 2A). With PC3 cells and PShTert myofibroblasts the gap closed more rapidly. After 96 hours, the PC3 cells had formed a distinct and much denser border of cells at the boundary of the two cell fronts, and appeared to invade through and clear the PShTert myofibroblasts. Where there were no PC3 cells, the PShTerts retained their morphology as observed in monoculture (Supplementary Figure 2B).

### PShTert-AR myofibroblasts induced PC3 cell apoptosis by paracrine signalling

**Figure 3:**
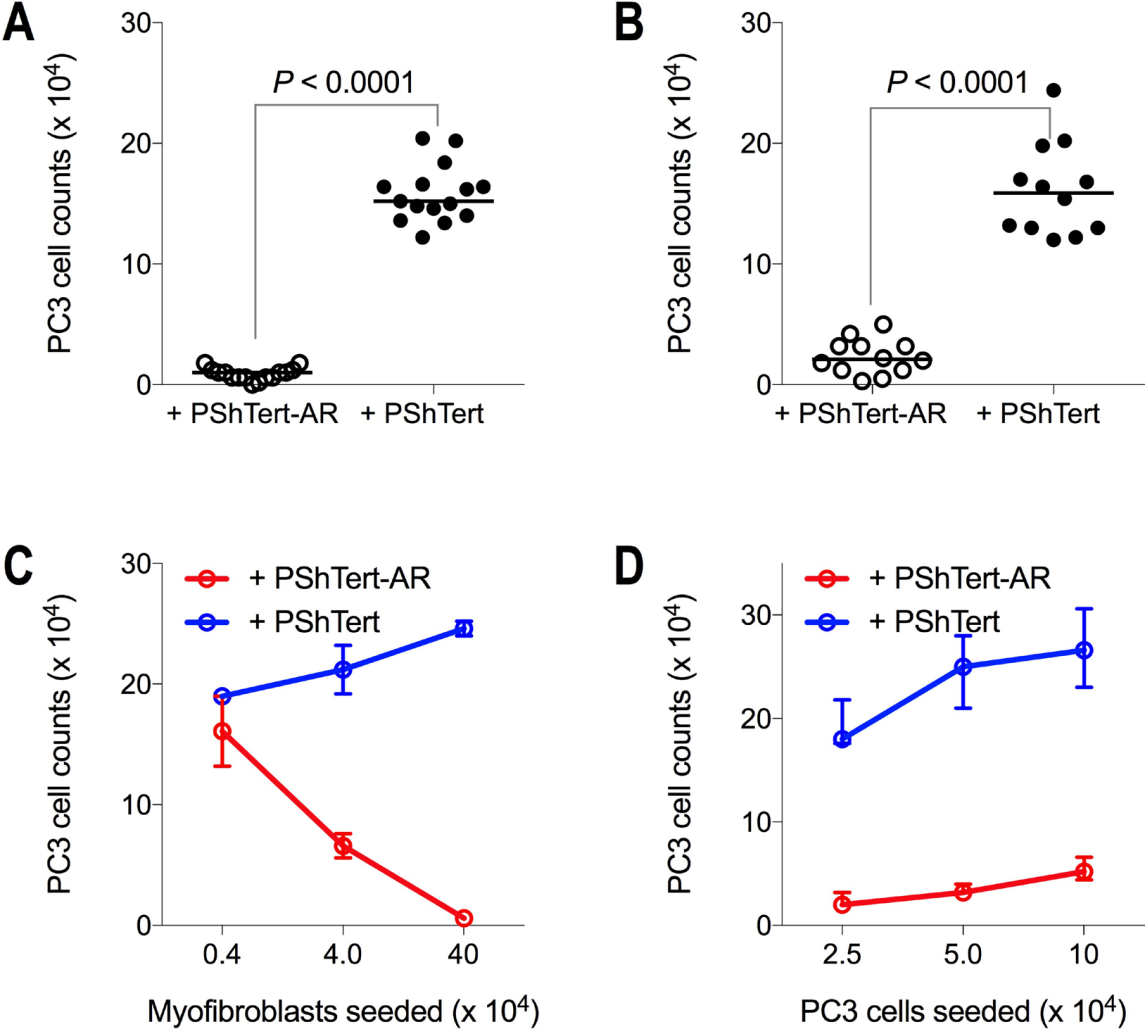
PC3 cell counts on day 6 of direct and indirect co-culture PC3 cells (5 × 10^3^) were either (**A**) directly or (**B**) indirectly co-cultured with PShTert-AR or PShTert myofibroblasts (4 × 10^5^). Medians of independent experiments shown; *n* = 15 (direct), *n* = 12 (indirect). *P*-values determined by Mann–Whitney *U*-test. (**C**) PC3 cells (5 × 10^3^) were directly co-cultured against decreasing numbers of myofibroblasts. (**D**) Increasing numbers of PC3 cells were directly co-cultured against a constant seeding density of myofibroblasts (4 × 10^5^). Medians with range shown of a single experiment performed in triplicate.

To investigate the nature of the signalling responsible for the changes observed in the cell growth, we compared cell counts in direct co-cultures to indirect co-cultures in transwell chambers. The results in Figure 3 show that after 6 days there were approximately 15-fold fewer PC3 cells following direct (Figure 3A) and indirect (Figure 3B) co-culture with PShTert-AR myofibroblasts compared to PShTert myofibroblasts. The PC3 cells in indirect co-culture were similar in morphology to those in direct co-culture.

We then investigated the effect of altering the seeding ratios of the two types of cells in the co-cultures to determine if this would influence the outcomes. Seeding a constant number of PC3 cells against decreasing numbers of myofibroblasts, revealed an inverse relationship between the number of PShTert-AR myofibroblasts seeded and the number of PC3 cells after 6 days of culture, but a direct relationship between the PShTert myofibroblasts and PC3 cells (Figure 3C). Increasing the number of PC3 cells seeded to a constant number of myofibroblasts did not alter the inhibitory effect of the PShTert-AR or the pro-proliferative effect of the PShTert myofibroblasts on the PC3 cell counts (Figure 3D). Thus, the ratio of myofibroblasts to PC3 cells influenced the degree, but not the nature, of the interactions between the co-cultured cells.

**Figure 4:**
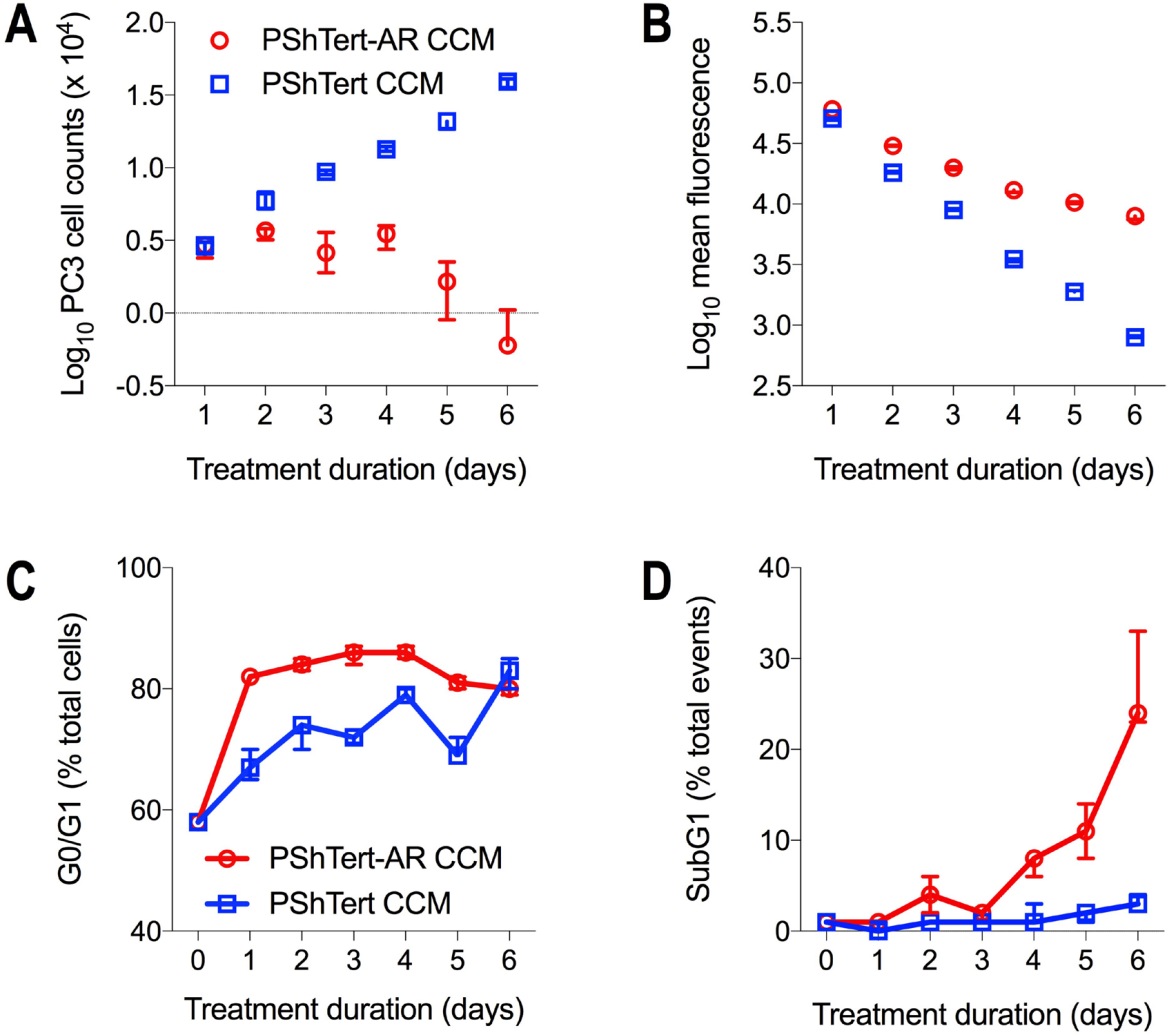
The effect of myofibroblast CCM on cell counts, proliferation and cell cycle PC3 cells (2.5 × 10^4^) were treated for 6 days with PShTert-AR or PShTert CCM replaced every 48 hours. PC3 cells were (**A**) counted and (**B**) CellTrace violet fluorescence intensity measured daily. For cell cycle analysis, PC3 cells (5 × 10^5^) were treated with myofibroblast CCM every 48 hours for 6 days. Cells were harvested and stained (25 μg/mL propidium iodide in DPBS containing 40 μg/mL RNase A) daily. (**C**) The percentage of total cells in G0/G1 of the cell cycle. (**D**) The percentage of total events in subG1. Data is the median and range of a single reproducible experiment.

The results from the indirect co-culture experiments suggested that paracrine factors from the PShTert-AR myofibroblasts were associated with the reduction in PC3 cell counts. We confirmed that the addition of PShTert-AR conditioned culture medium (CCM) to PC3 monocultures resulted in a significant reduction in PC3 cell numbers from day 3 onwards compared to cells grown in PShTert CCM (Figure 4A). The PC3 cells cultured with CCM from the myofibroblasts showed similar changes in cell morphology to those seen in co-cultures. These results showed that paracrine factors from the myofibroblasts were at least in part responsible for the changes observed in the PC3 cell morphology and number in co-culture.

**Figure 5:**
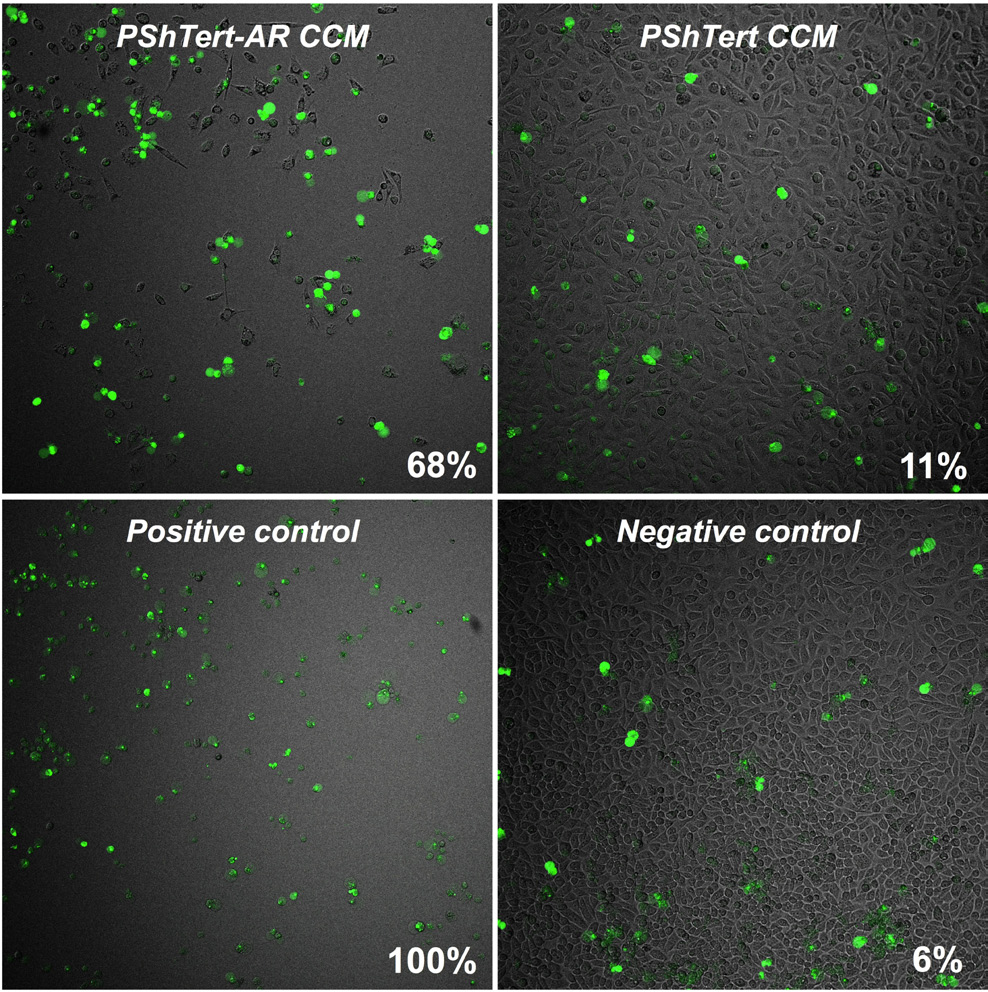
The effect of myofibroblast CCM on caspase-3/7 activity in PC3 cells Unlabelled PC3 cells (2.86 × 10^3^) were seeded overnight in μ-Plate 96-well plates (Ibidi) and treated with either PShTert-AR or PShTert CCM supplemented with CellEvent dye (1 μM). A positive control of PC3 cells treated with actinomycin D (200 nM) for 24 hours, and a negative control of PC3 cells in normal stripped medium, were also prepared with the inclusion of CellEvent. Cells were observed for 96 hours in real-time to detect the formation of a green fluorescence, indicative of activated caspase-3/7.

We investigated the mechanism for the reduction in PC3 cell numbers. There was a significant reduction in the rate of PC3 cell proliferation following treatment with CCM from PShTert-AR myofibroblasts, as evidenced by a reduction in the rate of dilution of CellTrace Violet fluorescence, evident from day 2 (Figure 4B). This was accompanied by an alteration in the cell cycle kinetics. There was an increase in the percentage of cells in G0/G1 from day 1 (Figure 4C), followed by a significant increase in subG1 events from day 4 onwards (Figure 4D). The latter was associated with a marked increase in the percentage of caspase-3/7 positive apoptotic cells (Figure 5). Together, these results show that CCM from the PShTert-AR myofibroblasts reduced PC3 cell numbers through inhibition of proliferation and induction of apoptosis.

### PC3 cells induced apoptosis in PShTert myofibroblasts in direct co-cultures

**Figure 6:**
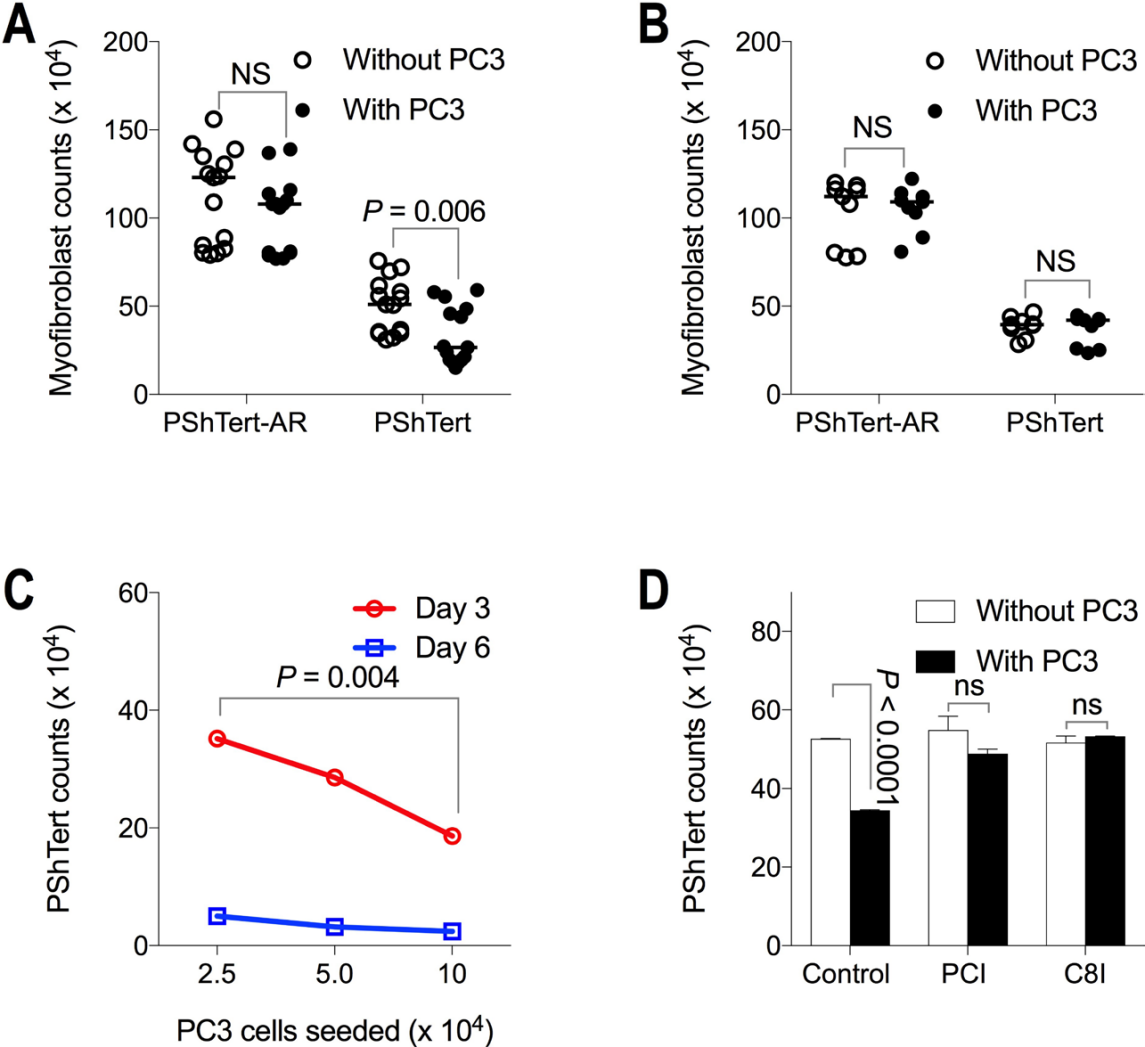
Myofibroblast counts in co-culture with PC3 cells Myofibroblast cell counts following 6 days of (**A**) direct and (**B**) indirect co-culture with PC3 cells compared to monoculture without PC3 cells. Medians of independent experiments shown; *n* = 15 (direct), *n* = 9 (indirect). *P*-values determined by Mann–Whitney *U*-test. (**C**) PShTert myofibroblast counts following 3 and 6 days of direct co-culture with PC3 cells of increasing seeding density. Medians and range from a single reproducible experiment. *P*-values calculated by unpaired, parametric Student’s *t*-test. (**D**) The effect of pan-caspase (PCI), and caspase-8 (C8I) inhibitors on PShTert myofibroblast counts in monoculture and direct co-culture with PC3 cells for 6 days. Medians and range of independent experiments; *n* = 2. *P*-values calculated by unpaired, parametric Student’s *t*-test.

Next, we investigated the destruction of the PShTert myofibroblasts by the PC3 cells in direct co-culture. There was a significant reduction in total PShTert myofibroblast counts in direct (Figure 6A), but not indirect (Figure 6B) co-culture, apparent microscopically from day 3. The number of surviving PShTert myofibroblasts in direct co-cultures with PC3 cells was inversely proportional to the PC3 cell seeding density (Figure 6C).

The loss of the PShTert myofibroblasts involved apoptosis. The myofibroblasts were positive for caspase-3/7 activation but only when in close proximity to PC3 cells (Supplementary Figure 3), and the loss of PShTert myofibroblasts in direct co-culture could be blocked almost completely by a pan-caspase inhibitor (PCI), and completely by a caspase-8 inhibitor (C8I) (Figure 6D). Together these results indicate that the myofibroblasts underwent apoptosis when in close contact with PC3 cells.

### Effects of the myofibroblasts occurred independent of prostate cancer cell AR expression

**Figure 7:**
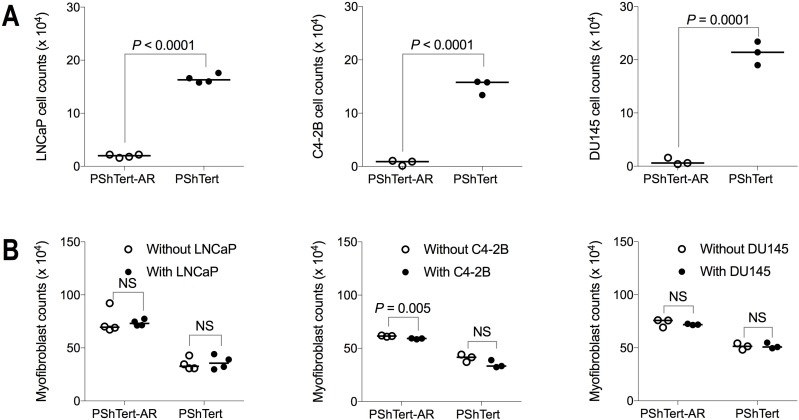
Cell counts after 6 days of direct co-culture between myofibroblasts and other prostate cancer cell lines PShTert myofibroblasts (4 × 10^5^) were directly co-cultured with, either LNCaP, C4-2B, or DU145 prostate cancer cell lines (5 × 10^3^) with cells harvested and counted on day 6. Cell counts for (**A**) prostate cancer cell lines and (**B**) myofibroblasts in monoculture and direct co-culture. Medians presented from a reproducible experiment. *P*-values calculated by unpaired, parametric Student’s *t*-test.

**Figure 8:**
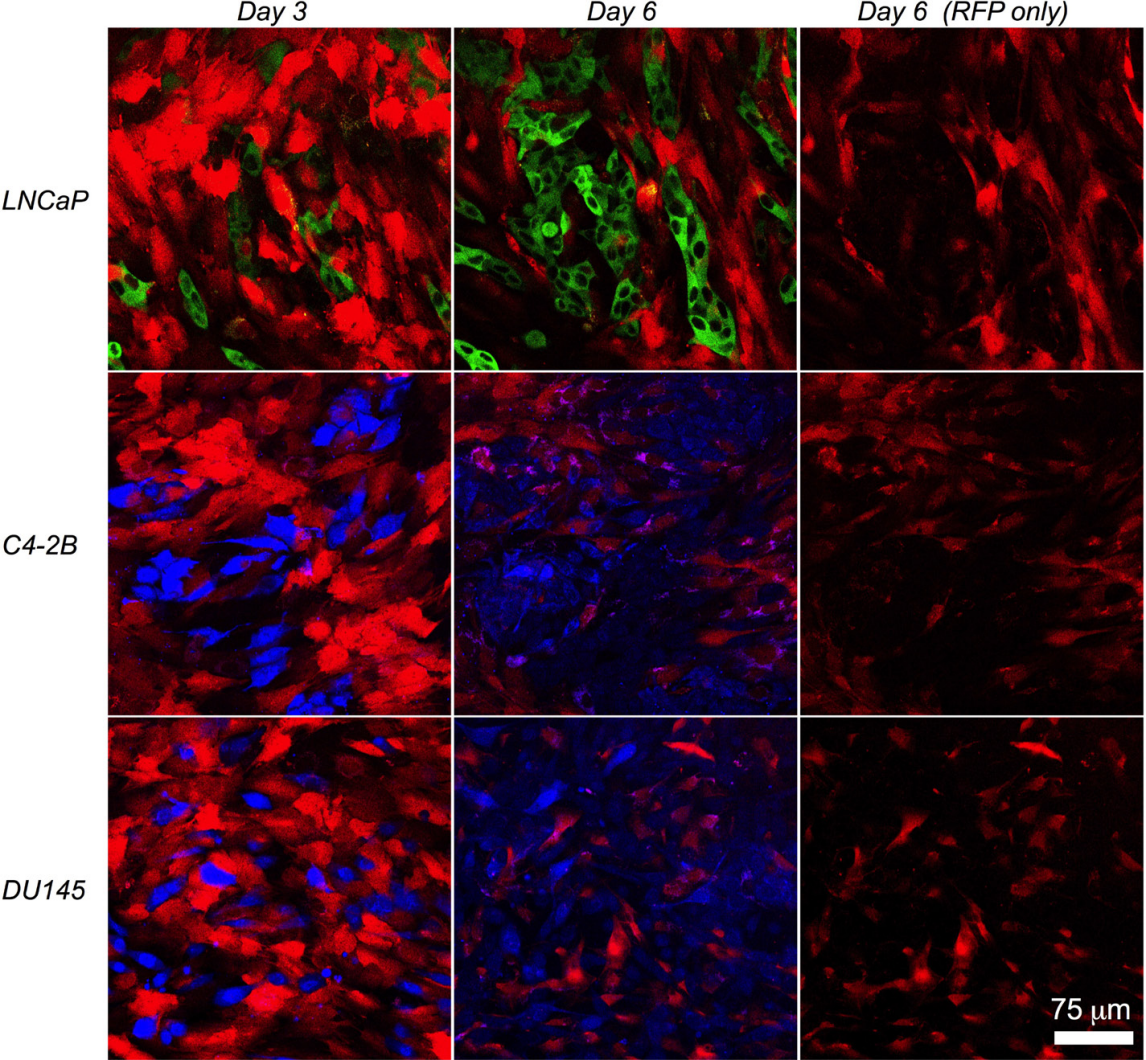
Morphology of PShTert myofibroblasts in direct co-culture with other prostate cancer cell lines PShTert myofibroblasts (red) were directly co-cultured with GFP-labelled LNCaP (green), or CellTrace Violet-labelled C4-2B, or DU145 prostate cancer cell lines (blue), with images captured on the LSM 700 in real-time for 6 days. Images represent morphology of PShTerts on day 3 and 6 of direct co-culture. Images on far right show the red channel (RFP) only for the day 6 images to show the morphological changes in PShTert myofibroblasts. Magnification 200×.

For most of our experiments the AR-negative PC3 prostate cancer cell line was used so that, of the cells in co-culture, only the myofibroblasts expressed AR. To determine if our observations were restricted to AR-negative PC3 cells, we set up direct co-cultures of the myofibroblasts with the AR-positive LNCaP and C4-2B, and the AR-negative DU145, prostate cancer cell lines. There was a significant reduction in the cell count of each of the prostate cancer lines when co-cultured with PShTert-AR myofibroblasts (Figure 7A). Whilst there was not a significant reduction in PShTert myofibroblast counts in direct co-culture with LNCaP, C4-2B or DU145 (Figure 7B), there was an obvious focal destruction of the PShTert myofibroblasts in the immediate proximity of these cancer cells (Figure 8). These results suggest that the effects of the myofibroblasts occurred independent of prostate cancer cell AR expression.

### 5α-dihydrotestosterone reduced PShTert-AR counts, which increased PC3 counts in co-culture

**Figure 9:**
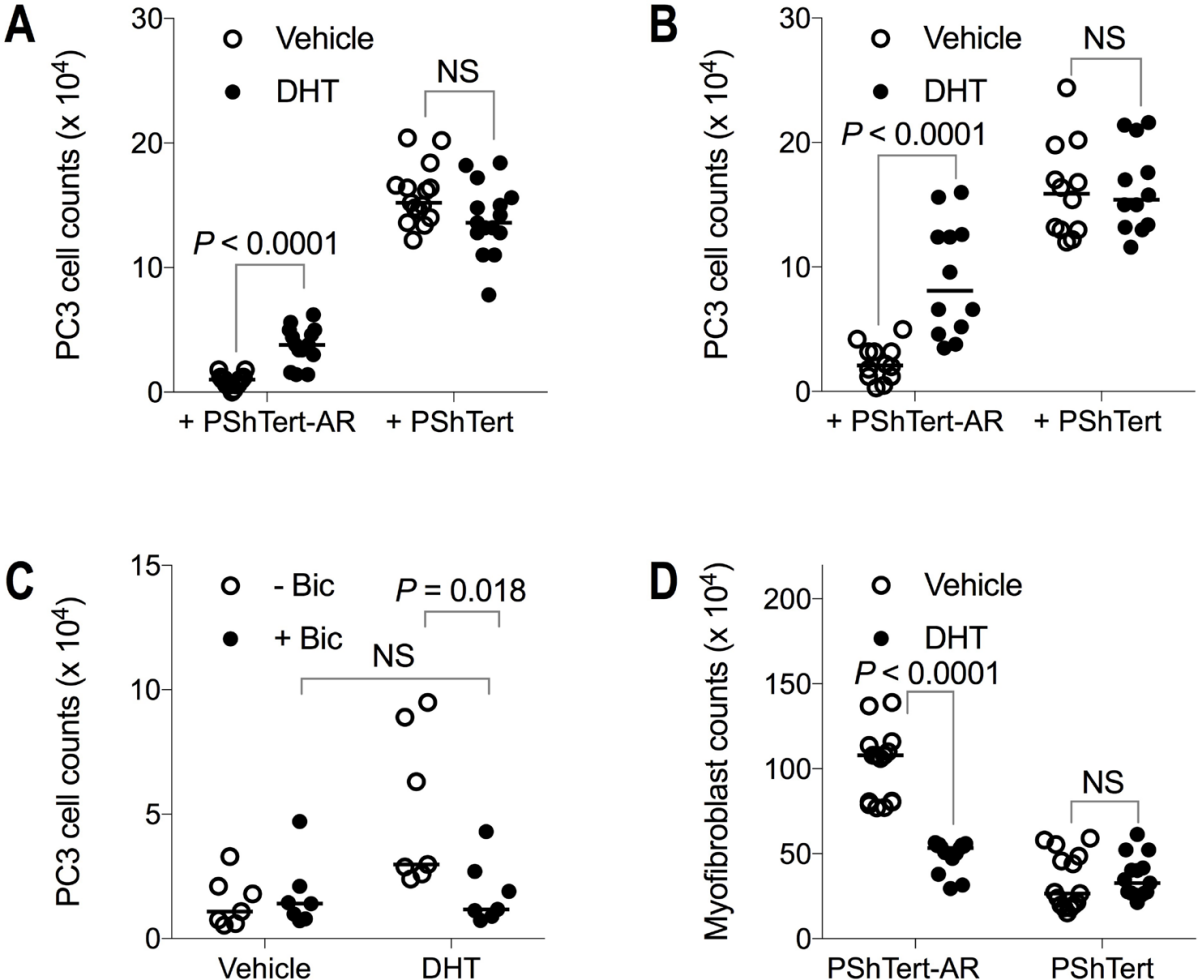
The effect of DHT on PC3 cell and myofibroblast counts in co-culture The effect of DHT on PC3 cell counts on day 6 of both (**A**) direct and (**B**) indirect co-culture with myofibroblasts. (**C**) Abrogation of the effect of DHT on PC3 cells indirectly co-cultured with PShTert-AR myofibroblasts by bicalutamide (*n* = 7). (**D**) The effect of DHT on myofibroblast counts (direct co-culture shown). Median values of multiple, independent experiments: (**A**) *n* = 15; (**B**) *n* = 12; (**C**) *n* = 7; and (**D**) *n* = 15. *P*-values calculated by Mann–Whitney *U*-test.

The results in Figure 9 show the effect of activation of the AR signalling pathway on the outcome of co-culture. The addition of the AR ligand 5α-dihydrotestosterone (DHT) to co-cultures with PShTert-AR myofibroblasts resulted in a significant 4-fold increase in the number of PC3 cells in both direct (Figure 9A) and indirect (Figure 9B) co-cultures. This increase in PC3 cell counts was abrogated by the anti-androgen bicalutamide in indirect co-culture (Figure 9C), confirming that DHT was acting through the AR signalling pathway in the myofibroblasts. DHT had no significant effect on PC3 cell counts in direct (Figure 9A) or indirect (Figure 9B) co-culture with PShTert myofibroblasts, consistent with the lack of AR in both of these cell types. The addition of DHT to myofibroblast monocultures resulted in a reduction in the number of PShTert-AR myofibroblasts over the period of culture, but no change in the number of PShTert myofibroblasts, as reported in a previous study [5]. In direct co-cultures treated with DHT there was also a significant reduction in the number of PShTert-AR myofibroblasts but not of PShTert myofibroblasts (Figure 9D). The focal destruction of the PShTert myofibroblasts observed adjacent to PC3 cells in direct co-cultures was not altered by the DHT. The higher recovery of PC3 cells with PShTert-AR in the presence of DHT, together with the results in Figure 3C, which show an inverse relationship between PShTert-AR and PC3 numbers in co-cultures, suggest that the increase in PC3 cell numbers was the result of a DHT induced decrease in the number of PShTert-AR myofibroblasts.

## DISCUSSION

The expression of AR in stromal fibroblasts is required for the development and maintenance of the normal prostate, and for the development of prostate cancer, yet interestingly stromal AR expression is frequently reduced in prostate cancer where it is associated with poor clinical outcomes [5]. Previously, we showed in a cohort of 64 patients that low AR expression is significantly associated with prostate cancer-related death at 1, 3, and 5 years post-diagnosis [5]. Others have also reported that the progressive loss of stromal AR correlates with progression of the disease, high-risk clinical parameters and/or poor outcome [3, 8, 13–16]. Why the loss of stromal AR is associated with poor outcome is unknown [6].

To address this question, we have studied the effect of AR expression in prostate myofibroblasts on the outcomes of direct and indirect co-culture with prostate cancer cells. We used hTERT immortalised myofibroblasts transduced with either AR (PShTert-AR), or empty expression vector (PShTert), in co-culture mostly with the AR-negative prostate cancer cell line PC3, so that we could isolate the effect of AR expression to the myofibroblast alone. Firstly, we observed a reduction in PC3 cell counts following direct or indirect co-culture with PShTert-AR myofibroblasts, compared to PShTert myofibroblasts. There was an inverse relationship between the numbers of PC3 cells recovered and the numbers of PShTert-AR myofibroblasts seeded. These effects were due to paracrine signals from the PShTert-AR myofibroblasts, which slowed the proliferation of the PC3 cells, with arrest at G0/G1, and increased their apoptosis. Secondly, we report the novel finding that direct but not indirect co-culture with PC3 cells significantly induced apoptosis in, and reduced the numbers of, PShTert myofibroblasts. The morphological changes and apoptosis were detected exclusively in PShTert myofibroblasts in contact with PC3 cells. In a confrontation assay the PShTert myofibroblasts promoted the migration and invasion of PC3 cells. Thirdly, we found that DHT reduced the proliferation of the PShTert-AR myofibroblasts, and, as a result of their reduced number, the number of PC3 cells increased.

Finally, we showed that the loss of cells in direct co-cultures occurred with other prostate cancer cell lines and irrespective of AR expression in those cells. The effects were observed not just with AR-negative PC3 cells, but also with AR-positive LNCaP and C4-2B, and AR-negative DU145 prostate cancer cell lines. Thus, the PShTert-AR myofibroblasts, in an androgen depleted environment, could control all cancer cell lines tested, whilst the PShTert myofibroblasts could not and were themselves destroyed.

Whilst a number of studies have investigated the interaction between fibroblasts and cancer cells in co-culture *in vitro*, most have compared different fibroblasts, such as normal versus cancer-associated, or different epithelial cells, such as normal versus malignant [17–26]. Few studies have compared prostate cancer myofibroblasts that differ in AR expression or signalling. The major difficulty is that within several passages *in vitro* primary human prostate myofibroblasts generally lose AR expression or do not express it at levels adequate to show androgen induced changes in gene expression [27]. One way to overcome this limitation is to stably transduce immortalised human prostate myofibroblasts with AR. This has been done previously using WPMY myofibroblasts transduced with either AR (WPMY-AR) or empty vector (WPMY-Vec). The conditioned medium from DHT-treated WPMY-AR cells significantly increased the growth of LNCaP prostate cancer cells, compared to conditioned medium from WPMY-Vec cells [27].

We explored both paracrine and direct signalling using an hTERT immortalised human prostate myofibroblast line, transduced with AR or empty vector. We have used the term direct signalling to describe the signalling which mediates the killing of the PShTert myofibroblasts by the cancer cells. We have shown that only those cells in very close proximity to the cancer cells are killed. We cannot distinguish between the killing by the cancer cells being mediated by paracrine signals that act only on immediately adjacent cells, or juxtacrine signals. We have used the term paracrine where the signalling can be demonstrated in a transwell chamber. To our knowledge, ours is the first *in vitro* study comparing the effect of myofibroblast AR expression or signalling on both direct and indirect interactions in prostate cancer.

The hTERT myofibroblasts we used are representative of cancer-associated fibroblasts (CAFs) and the PShTert-AR line has been shown to have a similar AR binding profile, and gene regulation, as primary fibroblasts and *in vivo* stroma [28]. Tissue recombination studies using these cell lines have produced results consistent with our *in vitro* findings. In nude male mice co-injected subcutaneously with PC3 cells and either PShTert-AR or PShTert myofibroblasts, tumour growth was reduced by PShTert-AR and promoted by PShTert [7]. Similarly, in castrated, immunodeficient NOD-SCID mice sub-renally grafted with a combination of human-derived primary prostate cancer tissue and either PShTert-AR or PShTert myofibroblasts, we found that grafts with PShTert-AR showed significantly more apoptosis in the cancer cells than grafts with PShTert [5]. Here we extend these *in vivo* studies by investigating the mechanistic basis for these observations *in vitro*.

We have shown that paracrine signalling by AR-expressing myofibroblasts slowed PC3 proliferation, and induced apoptosis *in vitro*. We have no indication whether the signalling moiety is molecular or exosomal. The death of cancer cells caused by fibroblasts has been reported by others, but not in the context of myofibroblast AR. In prostate cancer, conditioned culture medium, from bone marrow stromal cells, decreased the proliferation of and induced apoptosis in LNCaP and C4-2B, but not PC3 cells [29], CAFs induced apoptosis in gastric cancer cells [30], and human mesenchymal stem/stromal cells and CAFs, activated to express tumour necrosis factor (TNF)-alpha-related apoptosis-inducing ligand (TRAIL), induced apoptosis in breast cancer cells [31, 32]. Conversely, a number of other studies have reported that normal fibroblasts and/or CAFs inhibit cancer cell apoptosis [33–35]. None of these reports mentioned the fibroblast AR status.

Additionally, our results show that direct signalling was responsible for the destruction of the AR-negative myofibroblasts by apoptosis, with the effect that the PC3 cells were able to grow. The inability of these myofibroblasts to control the expansion of the cancer cells may explain why an AR-negative stroma is associated with more advanced prostate cancer. Several studies report observations consistent with ours, but not in the context of stromal AR. Normal human fibroblasts, in direct co-culture with prostate cancer cell lines PC3 and DU145, formed islands around the cancer cells early on and were eventually overtaken and almost completely destroyed by the growing cancer cells [26]. This arrangement of fibroblasts around tumour cells has also been described previously in direct co-cultures with HeLa cells [36], and in direct co-cultures of normal or malignant prostate epithelial cells with prostatic stromal cells from malignant tissue, where the epithelial cells displaced and grew within the stromal cells rather than growing on top [23, 35]. Breast cancer cells have been reported to release soluble factors that induced apoptosis in human bone marrow stromal cells *in vitro* [37], and lung fibroblasts were reduced in number, with evidence of apoptosis, following 3-dimensional co-culture with non-small cell lung cancer cell lines [38]. Another study reported that CAFs formed stromal islands in co-culture spheroids with prostate cancer cells, but were lost over time, with less then 10% remaining by day 8. The authors suggested that juxtacrine interactions were involved, but the mechanisms were not investigated, and, although they mentioned the CAFs were AR-negative, they did not explore whether similar effects occurred with AR-positive CAFs [39]. Here, we have confirmed that direct signalling was responsible for the loss of the AR-negative myofibroblasts, through the induction of apoptosis, with no loss of myofibroblasts that expressed AR.

We have shown that myofibroblasts stably transduced with AR prevented the growth of prostate cancer cells, even though the experiments were performed in stripped media which has no, or a very low, concentration of androgen. The growth inhibitory effect was partially ablated by the addition of DHT. This suggests that it is the expression of AR, not AR signalling, which results in the phenotypic and functional differences noted in the PShTert-AR myofibroblasts compared to those transduced with empty vector. This conclusion is consistent with other reports. The stable transduction of AR into WPMY human prostate myofibroblasts significantly altered their gene expression pattern compared to those transduced with empty vector, in the absence of DHT [27]. Knockdown of AR by siRNA in an AR-positive cancer-associated fibroblast line produced significant differences in the expression of several growth factor genes, and the proliferation and migration of PC3 cells in transwell co-cultures [40], and the transfection of human AR into AR-deficient mouse Sertoli cells significantly altered the expression of 672 genes in the absence of androgen stimulation [41]. These latter two studies did not specify whether stripped medium was used. Together, these studies provide strong evidence that there are ligand independent effects from AR expression in prostate cancer myofibroblasts. Whilst we determined this by comparing the effect of the same myofibroblast line transduced either with AR or empty vector, a potential limitation of our study is that we did not determine the effect of the knockdown of myofibroblast AR. This could have provided further confirmation that the findings were due to AR expression and not other potential differences between the myofibroblast lines.

Here we have shown that the outcomes from co-culturing human prostate myofibroblasts and prostate cancer cell lines, either AR-positive or AR-negative, differ between myofibroblasts that express or lack AR, and involve paracrine and direct signalling. These studies are consistent with the clinical findings that loss of stromal AR is associated with reduced survival in prostate cancer. The findings suggest that AR-expressing myofibroblasts inhibit prostate cancer progression through paracrine signals that slow proliferation and induce apoptosis in the cancer cells. In contrast, myofibroblasts lacking AR expression permit prostate cancer progression, as they do not inhibit cancer cell proliferation or migration and undergo apoptosis when in close contact with the cancer cells. Our findings suggest that a better understanding of the regulation and function of AR expression in stromal myofibroblasts, and of the interactions between the cancer cells and stromal myofibroblasts, will increase our understanding of the biology of indolent and aggressive prostate cancers, and may lead to the development of novel treatments which can modify their progression.

## METHODS

### Cell lines and cell culture

The prostate cancer cell lines PC3, LNCaP, C4-2B and DU145 were obtained from the American Type Culture Collection (Manassas, VA, USA) and were maintained in complete RPMI consisting of RPMI 1640 (Life Technologies, Grand Island, NY, USA) supplemented with 10% fetal bovine serum (FBS; Sigma-Aldrich, St Louis, MO, USA), 200 U/mL penicillin and 200 μg/mL streptomycin (Life Technologies). Telomerase immortalised human prostate stromal myofibroblasts stably transduced with AR (PShTert-AR) or empty vector (PShTert) were obtained from Professor Peng Lee, Department of Pathology, New York University School of Medicine [7], and were maintained in DMEM (Life Technologies) supplemented with 10% FBS, 200 U/mL penicillin and 200 μg/mL streptomycin. All cell lines were authenticated via Short Tandem Repeat testing in 2014 or 2016 (DU145), by CellBank Australia (NSW, Australia) [5, 42], and were cultured at 37° C with 5% CO_2_ in air.

### Fluorescent labelling of cell lines

Luciferase-tagged PC3 and LNCaP cells were generated to express green fluorescent protein (GFP) by stably transducing with the triple reporter gene construct SFG-NES-TGL as described previously [43] and were a kind gift from Professor Andreas Evdokiou. The C4-2B and DU145 cells were labelled using the CellTrace Violet (CTV) Cell Proliferation Kit according to the manufacturer’s protocol (Life Technologies). The PShTert-AR and PShTert myofibroblasts were stably transduced with the SFG-RFP/Rluc construct to express red fluorescent protein (RFP) as described previously [44].

### Western immunoblot analysis

The AR expression of each of the cell lines was confirmed by western immunoblot as previously described [45].

### Direct/indirect co-cultures and confrontation assays

RFP-labelled myofibroblasts were cultured for 24 hours in phenol red free RPMI 1640 containing L-glutamine (Life Technologies), supplemented with 10% dextran-coated charcoal-stripped FBS (Equitech-Bio, Inc., Kerrville, TX, USA), 200 U/mL penicillin and 200 μg/mL streptomycin (stripped medium), then seeded in stripped medium into six-well plates (BD Biosciences, San Jose, CA, USA), or dishes with imprinted cell relocation grid (μ-Dish 35 mm, Grid-500; Ibidi, Martinsried, Germany), and incubated for 48 hours. Labelled prostate cancer cells resuspended in stripped medium were either seeded onto the myofibroblast monolayer for direct co-culture, or onto polyester membrane inserts, with 0.4 μm pores (Corning Inc. Life Sciences, Tewksbury, MA, USA), placed in wells of myofibroblast monolayers, for indirect co-culture. The medium was replaced with fresh, stripped medium on day 3 of co-culture. To test the effect of androgen on the cultures, either vehicle (0.1% ethanol), 10 nM 5α-dihydrotestosterone (DHT; Sigma-Aldrich), 10 μM bicalutamide (Bic; Sigma-Aldrich), or 10 nM DHT and 10 μM Bic were added at the time that the myofibroblasts were seeded into wells (day –2), on the addition of the PC3 cells (day 0), and on day 3 of co-culture. Confrontation assays between myofibroblasts and PC3 cells were prepared by seeding the cells in separate chambers (500 μm apart) of the Ibidi Culture-Insert 2 well positioned in an Ibidi μ-Dish 35 mm (3.5 × 10^4^ cells per well). Cells were left to adhere for 16 hours under standard culture conditions. Culture inserts were carefully removed and the cells washed with Dulbecco’s phosphate buffered saline (DPBS; Life Technologies) 3 times followed by replacement with stripped medium. The interface where the two cell types met as they proliferated and migrated was monitored by time-lapse fluorescence microscopy.

### Morphological evaluation

Cell morphology was assessed daily by fluorescence microscopy using an Axio Observer.Z1 with HBO 100 illuminator and AxioVision Rel 4.8 software (Carl Zeiss Microscopy GmbH, Jena, Germany). High-power images were acquired using a LSM 700 confocal microscope with Zen software (Zeiss).

### Cell counts

Cells were washed with DPBS, incubated with 0.25% trypsin-EDTA (Life Technologies), and resuspended in stripped medium. Cells were centrifuged at 300 × g for 5 minutes, and resuspended in DPBS. Fluorescently labelled cells were counted using a haemocytometer under fluorescence microscopy.

### Preparation of myofibroblast conditioned culture medium

RFP-labelled PShTert-AR or PShTert myofibroblasts were cultured for 24 hours in stripped medium, and then seeded into flasks in stripped medium at 7.2 × 10^6^ cells per 175 cm^2^. Conditioned culture medium (CCM) was collected and replaced with fresh, stripped medium every 2 days for 6 days.

### Cell proliferation

GFP-labelled PC3 cells were labelled using the CTV Proliferation Kit, seeded in stripped medium at 2.5 × 10^4^ cells per well in six-well plates, and incubated for 5 hours until the cells were adherent. The medium was replaced with freshly prepared myofibroblast CCM every 2 days for up to 6 days. Cells were harvested every day for 6 days, washed, and resuspended in DPBS. Cell counts were performed and the CTV fluorescence intensity was determined using a FACSCanto II flow cytometer (BD Biosciences, Franklin Lakes, NJ, USA). Cell doublets were excluded by doublet discrimination, based on non-linearity of forward scatter and side scatter area versus height plots. Proliferation was quantitated by dye dilution.

### Cell cycle analysis

GFP-labelled PC3 cells were seeded in stripped medium at 5 × 10^5^ cells per well in six-well plates, and incubated for 24 hours. The medium was replaced with freshly prepared myofibroblast CCM every 2 days for 6 days. Cells were harvested every day for 6 days, washed, resuspended in DPBS, and fixed with a final concentration of 70% ice cold ethanol. Next, cells were pelleted, rehydrated with 0.25% Triton X-100 in DPBS, and stained for 2 hours with 25 μg/mL propidium iodide in DPBS containing 40 μg/mL RNase A. Cells were analysed using a FACS Canto II flow cytometer, with doublets excluded. Cells in G0/G1 and G2/M were calculated as the percentage of total cells (i.e., total events minus subG1 events). The subG1 population was calculated as the percentage of total events.

### Investigating caspase-3/7 activity and cell death pathways

To measure apoptosis induced by myofibroblast CCM, unlabelled PC3 cells were seeded at 2.86 × 10^3^ cells per well in μ-Plate 96-well plates (Ibidi) and cultured overnight. The medium was replaced with either stripped medium or fresh CCM supplemented with 1 μM CellEvent Caspase-3/7 Green Detection Reagent (Life Technologies).

To measure apoptosis in direct co-cultures, RFP-labelled PShTert myofibroblasts in stripped medium were seeded at 1.1 × 10^4^ cells per well in μ-Plate 96-well plates and cultured for 2 days. Next, 1.43 × 10^3^ GFP-labelled PC3 cells per well in stripped medium supplemented with 1 μM CellEvent Caspase-3/7 Green Detection Reagent were added directly onto the myofibroblast monolayer. Cells treated with 200 nM actinomycin D (Sigma-Aldrich) were used as a positive control. Cells were monitored for 5 days using a LSM 700 confocal microscope. The mean percentage of apoptotic cells was determined from two high-power fields of view. To measure the effect of caspase inhibition, PShTert myofibroblasts (4 × 10^5^) were seeded for 48 hours and then overlaid with medium containing either no cells or PC3 cells (5 × 10^3^), and supplemented with either vehicle (0.1% dimethyl sulfoxide; Sigma-Aldrich), a pan-caspase inhibitor (PCI; Z-VAD-FMK; 20 μM; Calbiochem Merck, Darmstadt, Germany), or a caspase-8 inhibitor (C8I; Z-IETD-FMK; 20 μM; R&D Systems, Minneapolis, MN, USA). Actinomycin D (200 nM) was used with the pan-caspase inhibitor as a positive control. The medium was replaced on day 3 and the cells counted on day 6.

### Data analysis

All graphs and statistical analyses were generated using GraphPad Prism version 6.0d (GraphPad software Inc., San Diego, CA). Unless otherwise indicated, groups were compared using student *t*-tests, and differences were considered significant when *P*-values were ≤0.05.

### Data availability

All data generated or analysed during this study are included in this published article (and its Supplementary Information files).

## SUPPLEMENTARY MATERIALS FIGURES



## Abbreviations

AR: androgen receptor
CAF: cancer-associated fibroblast
C8I: caspase-8 inhibitor
CTV: CellTrace Violet
CCM: conditioned culture medium
DHT: 5α-dihydrotestosterone
DPBS: Dulbecco’s phosphate buffered saline
FBS: fetal bovine serum
GFP: green fluorescent protein
PCI: pan-caspase inhibitor
RFP: red fluorescent protein
TNF: tumour necrosis factor
TRAIL: TNF-alpha-related apoptosis-inducing ligand
