# Supplementary material for: Myofibroblast androgen receptor expression determines cell survival in co-cultures of myofibroblasts and prostate cancer cells in vitro
Source: Oncotarget. 2018 Apr 10;9(27):19100–14. doi: 10.18632/oncotarget.24913 (PMC5922380; doi:10.18632/oncotarget.24913)
Supplement: Supplementary file 1 [file oncotarget-09-19100-s001.pdf]

Myofibroblast androgen receptor expression determines cell survival in co-cultures of myofibroblasts and prostate cancer cells *in vitro*

SUPPLEMENTARY MATERIALS

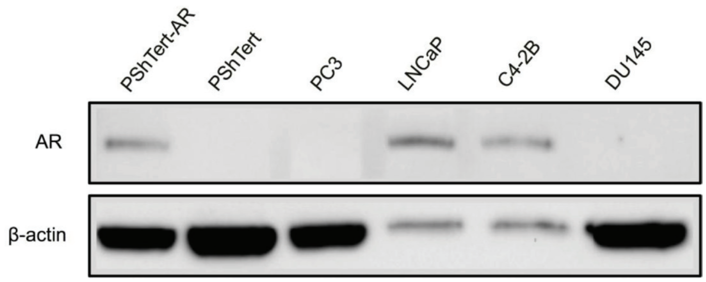

Supplementary Figure 1: Western immunoblot for AR expression and the housekeeping protein β-actin in myofibroblast lines PShTert-AR and PShTert, and prostate cancer cell lines PC3, LNCaP, C4-2B and DU145.

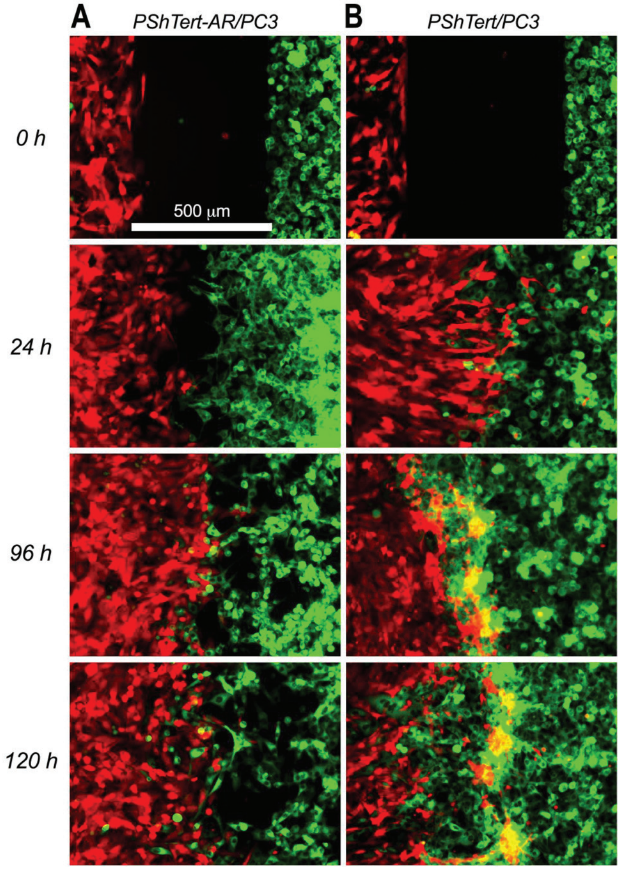

Supplementary Figure 2: Confrontation assay between (A) PShTert-AR or (B) PShTert myofibroblasts and PC3 cells. PC3 cells and myofibroblasts were seeded in separate wells of an Ibidi Culture-Insert 2 well (3.5×10^4 cells per well) and left to adhere. Culture inserts were removed, cells washed and medium replaced (0 hours). Images of the 500 μm gap were captured at 0, 24, 96, and 120 hours to monitor movement of the two cell fronts. Original magnification 100×.

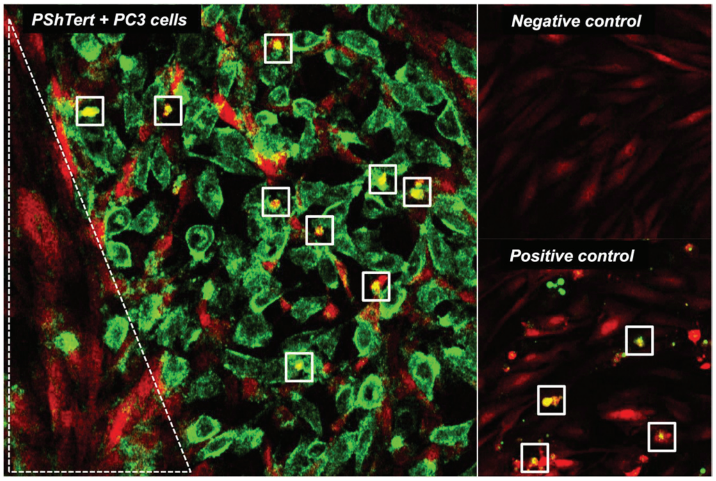

Supplementary Figure 3: Caspase-3/7 activation in PShTert myofibroblasts in direct contact with PC3 cells. PShTert myofibroblasts (1.1×10^4) were directly co-cultured with PC3 cells (1.43×10^3) in normal medium supplemented with CellEvent (1 µM). Cells were monitored in real-time for 96 hours. Caspase-3/7 was activated only in PShTert myofibroblasts in contact with PC3 cells (squares). The area of PShTert myofibroblasts devoid of PC3 cells (dotted line) showed no evidence of caspase-3/7 activation. PShTert myofibroblasts treated with normal stripped medium or medium supplemented with actinomycin D (200 nM), both with CellEvent, were used as negative and positive controls respectively.
